# Treating juvenile idiopathic arthritis to target: what is the optimal target definition to reach all goals?

**DOI:** 10.1186/s12969-020-00428-7

**Published:** 2020-04-16

**Authors:** Casper G. Schoemaker, Joost F. Swart, Nico M. Wulffraat

**Affiliations:** 1 Department of Pediatric Rheumatology and Immunology, Wilhelmina Children’s Hospital, University Medical Center Utrecht, Room KC.03.063.0, P.O. box 85090, 3508 AB Utrecht, The Netherlands; 2 Faculty of Medicine, Utrecht University, Utrecht, The Netherlands; 3 Netherlands JIA Patient and Parent Organisation, member of ENCA, Rijssen, The Netherlands

**Keywords:** Juvenile idiopathic arthritis, Treatment, Disease activity, Patient perspective, Treat to target, Fatigue, Pain, Stiffness, Shared decision making

## Abstract

In 2018, an international Task Force formulated recommendations for treating Juvenile Idiopathic Arthritis (JIA) to target. The Task Force has not yet resolved three issues. The first issue is the lack of a single “best” target. The Task Force decided not to recommend the use of a specific instrument to assess inactive disease or remission. Recent studies underscore the use of a broad target definition. The second issue is the basic assumption that a treatment aggressively aimed at the target will have ‘domino effects’ on other treatment goals as well. Thus far, this assumption was not confirmed for pain, fatigue and stiffness. The third issue is shared decision-making, and the role of individual patient targets. Nowadays, patients and parents should have a more active role in choosing targets and their personal treatment goals. In our department the electronic medical records have been restructured in such a way that the patient’s personal treatment goals with a target date appears on the front page. The visualization of their specific personal goals helps us to have meaningful discussions on the individualized treatment strategy and to share decisions. In conclusion, a joint treat to target (T2T) strategy is a promising approach for JIA. The Task Force formulated valuable overarching principles and a first version of recommendations. However, implementation of T2T needs to capture more than just inactive disease. Patients and parents should have an active role in choosing personal targets as well.

In 2018, an international Task Force formulated recommendations for treating Juvenile Idiopathic Arthritis (JIA) to target [1]. The Task Force addressed specific treatment targets and described the Treat to Target (T2T) strategy to reach the therapeutic goals. Their recommendations were intended to provide expert guidance on general treatment approaches to improve patient care in standard clinical practice. In this commentary, we will elaborate on three issues that have not been resolved by the Task Force: the target definition, the assumption that reaching the target will have effects on the other goals as well, and the role of individual patient targets in shared decision-making.

The first issue is the lack of a single ‘best’ target. Although most would agree that clinical inactive disease (CID) or remission is the ultimate target, there are multiple ways in which this disease state can be assessed in the clinical setting [2–6]. Wallace’s preliminary criteria capture more objective measures of inflammation, and can be regarded a narrow target [7]. The clinical Juvenile Arthritis Disease Activity Score (cJADAS), through inclusion of a global measure of patient well-being, may also capture other non-inflammatory components of the disease, such as chronic pain and fatigue and is regarded a broader target [4, 8]. The Task Force decided not to recommend the use of a specific instrument, leaving the choice to the clinician [1].

Two studies found evidence in favour of the broader target in T2T strategies. Swart et al. concluded that parental overall scores of well-being increased the identification of methotrexate (MTX) non-responders [8]. The omission of the patient visual analogue scale (VAS) well-being scores in the cJADAS prognostic test resulted in a decreased identification of MTX non-responders. Patients and parents take into account all complaints such as morning stiffness, fatigue, and joint pain on every occasion between the visits and not merely the active joint count at one visit. The cJADAS incorporates the patient global VAS, is very user-friendly and does not require erythrocyte sedimentation rate (ESR) results before a decision can be made [8]. Guzman et al. found that in a T2T strategy for JIA, pain experience by the patient in the last week and overall parental assessment added to the model to predict remission [9]. These findings underscore the use of a broad target definition.

The second issue we would like to address is that reaching the target – whether it is CID or remission – is not the one and only goal of T2T in JIA. Indeed, according to the Task Force there are several goals for treating patients with JIA to target: “to control signs and symptoms; to prevent structural damage; to avoid comorbid conditions and drug toxicities; and to optimise function, growth and development, quality of life, and social participation” [1]. The main assumption of a T2T-approach is that a treatment aggressively aimed at the target will have ‘domino effects’ on all other goals as well [10–12]. In recent studies, this assumption has been tested for T2T in JIA. Here we will lay out their results for three important patient goals: pain, fatigue and stiffness [13].

Bromberg et al. reported that self-reported pain, fatigue and stiffness continue to be common in JIA patients, despite contemporary advances in treatment strategies, including biological agents that are able to induce clinical inactive disease [14, 15]. In these patients, aggressive treatment strategies did affect disease activity, but not chronic pain. In a longitudinal study, Anink et al. reported that although disability and disease activity were low, chronic pain remained an issue, 8 years after the start of etanercept treatment [16]. As Lomholt put it: “pain was still a problem for a subgroup of children though they were in remission with biological agents” [17]. In a trajectory analysis, Shiff et al. reported that disease activity only accounted for a small portion of variability in pain severity in children with JIA [18]. The same picture emerged for fatigue: although fatigue is a prominent feature in many JIA patients, disease activity seems not to be directly related to it [19–21]. Interestingly, Ringold et al. hypothesized that pain may be an intermediate link between disease activity and fatigue [21]. More trajectory analyses of these complex domino-effects are clearly needed .

Thus far, the assumptions that treat to target strategies will ultimately lead to less pain, fatigue and stiffness in all patients is not confirmed. In terms of best outcomes for the child, these results highlight the importance of addressing all aspects of JIA and not just the underlying inflammation [2, 15, 22]. Treating to target in JIA requires a multifactorial treatment strategy, potentially including interventions such as physiotherapy and early pain/fatigue coping strategies delivered by a multidisciplinary team for children with fatigue or chronic pain in the absence of joints with overt active disease [2, 14, 23].

The third unresolved issue is shared decision-making, and the role of individual patient targets. “The treatment targets and the therapeutic strategy should be based on shared decisions between the parents/patient and the paediatric rheumatology healthcare team,” is one of the overarching principles of the Task Force [1]. In their explanation, the Task Force described shared decision making as follows: “the parents/patient must be informed about and agree with the selected target, the therapeutic options to reach the target and the reason for choosing the target, also in the light of the risks related to both the treatment and the disease”. We strongly believe that nowadays, patients and parents could and should have a more active role in choosing targets and their personal treatment goals [24–26]. Ultimately, this will lead to better treatment adherence.

In the Pediatric Rheumatology and Immunology department of the Wilhelmina Children’s Hospital the electronic medical records have been restructured in such a way that the patient’s personal treatment goals with a target date appears on the front page. In our experience, many patients formulate their own goals for symptoms like fatigue and pain. Interestingly, they tend to formulate social goals as well: participation in social activities at school, sports, or with friends. In their personal goals we recognize the important themes in living with JIA as reported in qualitative studies [27, 28]. In our experience, disease activity per se is an outcome that patients and parents may not fully comprehend, although a graphical display with target lines of minimal disease activity of the cJADAS does provide more insight [8]. However, the visualization of their specific personal goals helps us to have meaningful discussions on the individualized treatment strategy and to share decisions. Reaching the patients’ own goal is always a special moment to celebrate in the consulting room, while formulating the next one if needed. Since the introduction of the possibility of setting personal treatment goals in the electronic medical records, patients and parents are more engaged in the treatment decisions as well, while physicians better understand what matters most to the patient. Everybody is now fully aware of which goal we want to reach at the set date and if we are close to reaching it or not at all, the latter requiring more effort. Furthermore we believe that setting personal future goals already at onset of the disease might prevent to settle for less when time passes. The resilience and acceptance of status quo by patients and parents is admirable, but should never lead to a quality of life less than the highest possible one.

## Conclusion

We do believe that a joint T2T strategy is a promising approach for JIA. The Task Force formulated valuable overarching principles and a first version of recommendations. Recent studies question the assumption that reaching remission in T2T will lead to attaining the downstream goals as well. Implementation of T2T therefore needs to capture more than just inactive disease. Patients and parents should have an active role in choosing personal targets as well. In this respect, the lack of participation of parent or patient representatives was a limitation of the work of the Task Force thus far [29, 30]. Fortunately, the Task Force insisted that with the next iteration, parents and patients will be included [1].

## Data Availability

Not applicable.

## Abbreviations

CID: clinical inactive disease
cJADAS: clinical Juvenile Arthritis Disease Activity Score
ESR: erythrocyte sedimentation rate
JIA: Juvenile Idiopathic Arthritis
MTX: methotrexate
T2T: treat to target
VAS: visual analogue scale
